# Bacterial infection systemically suppresses stomatal density

**DOI:** 10.1111/pce.13570

**Published:** 2019-06-10

**Authors:** Christian Dutton, Hanna Hõrak, Christopher Hepworth, Alice Mitchell, Jurriaan Ton, Lee Hunt, Julie E. Gray

**Affiliations:** ^1^ Department of Molecular Biology and Biotechnology University of Sheffield Sheffield S10 2TN UK; ^2^ Department of Animal and Plant Sciences University of Sheffield Sheffield S10 2TN UK; ^3^ Grantham Centre for Sustainable Futures University of Sheffield Sheffield S10 2TN UK

**Keywords:** Arabidopsis thaliana, flagellin receptor, lipid transfer protein, plant pathogen, *Pseudomonas syringae*, salicylic acid, stomatal development, systemic signal

## Abstract

Many plant pathogens gain entry to their host via stomata. On sensing attack, plants close these pores to restrict pathogen entry. Here, we show that plants exhibit a second longer term stomatal response to pathogens. Following infection, the subsequent development of leaves is altered via a systemic signal. This reduces the density of stomata formed, thus providing fewer entry points for pathogens on new leaves. Arabidopsis thaliana leaves produced after infection by a bacterial pathogen that infects through the stomata (*Pseudomonas syringae*) developed larger epidermal pavement cells and stomata and consequently had up to 20% reductions in stomatal density. The bacterial peptide flg22 or the phytohormone salicylic acid induced similar systemic reductions in stomatal density suggesting that they might mediate this effect. In addition, flagellin receptors, salicylic acid accumulation, and the lipid transfer protein AZI1 were all required for this developmental response. Furthermore, manipulation of stomatal density affected the level of bacterial colonization, and plants with reduced stomatal density showed slower disease progression. We propose that following infection, development of new leaves is altered by a signalling pathway with some commonalities to systemic acquired resistance. This acts to reduce the potential for future infection by providing fewer stomatal openings.

## 1 INTRODUCTION

Gaseous diffusion between plants and the atmosphere is limited by the size and density of stomatal pores in the leaf epidermis. Most plant species can adjust their stomatal development to suit the environmental conditions. Light intensity, carbon dioxide (CO_2_) concentration, and relative humidity all influence the frequency of stomatal initiation in developing leaves (Lake, Quick, Beerling, & Woodward, 2001; Schoch, Zinsou, & Sibi, 1980; Thomas, Woodward, & Quick, 2004; Woodward, 1987). Exposure of mature leaves to elevated concentrations of CO_2_ results in new leaves with reduced stomatal density (SD) and stomatal index (SI; the ratio of stomata to epidermal cell number; Lake et al., 2001). Similarly, the light intensity experienced by mature leaves systemically affects SI and SD of new leaves (Lake et al., 2001; Schoch et al., 1980; Thomas et al., 2004). These observations suggest that long distance systemic signal(s) travel from mature leaves to leaf initials, where they influence cell fate decisions and modulate epidermal development. The molecular processes leading to formation of stomata in developing organs and how stomatal development might be attenuated by abiotic signals have been extensively studied (for recent reviews, see Chater, Oliver, Casson, & Gray, 2014; Han & Torii, 2016; Simmons & Bergmann, 2016). For example, the epidermal patterning factor EPF2 is involved in adjusting SD in response to changing CO_2_ levels (Engineer et al., 2014). However, little is known about the long distance signals responsible for systemically modulating stomatal development in newly developing leaves. Although stomata are important for photosynthesis, they represent vulnerable sites that are exploited for entry by a range of foliar pathogens. Pathogenic bacteria, such as *Pseudomonas syringae* pv. *tomato* DC3000 (*Pst*DC3000), colonize the leaf surface and aggregate around the stomatal pores, where they gain entry.

Stomata are not passive entry points but can close to restrict pathogen entry after detection of conserved molecular patterns (Melotto, Underwood, Koczan, Nomura, & He, 2006). Stomatal closure forms part of the plant innate immune response that is commonly referred to as pattern‐triggered immunity (PTI). In addition to localized stomatal closure responses, PTI controls the production of long‐distance signals that systemically prime plants against future attack, resulting in systemic acquired resistance (SAR; Bigeard, Colcombet, & Hirt, 2015; Conrath et al., 2006; Jung, Tschaplinski, Wang, Glazebrook, & Greenberg, 2009). Two previous studies have revealed a link between Arabidopsis systemic stomatal development and pathogen infection; powdery mildew causes increases in both SD and SI, whereas leaves developing after viral infection have decreased SD and SI (Lake & Wade, 2009; Murray, Emblow, Hetherington, & Foster, 2016). However, neither of these pathogens infect through the stomatal pores, and the mechanisms as well as the potential benefits of these responses to either the plant or pathogen remain unclear. The aim of the current study was to determine whether infection by bacterial pathogens that enter via stomata could systemically affect stomatal development and whether altered stomatal development could, in turn, affect susceptibility to these pathogens.

To date, several long‐distance SAR signals have been described in Arabidopsis (Dempsey & Klessig, 2012; Liu, von Dahl, Park, & Klessig, 2011), and we investigated whether these could also be involved in the systemic regulation of SD. These include the lipid derivative azelaic acid (AzA), which accumulates in petiole exudates of infected Arabidopsis leaves and is transported to systemic leaves (Jung et al., 2009); pipecolic acid (L‐Pip), which accumulates in infected leaf exudates and distal leaves (Návarová, Bernsdorff, Döring, & Zeier, 2012); and its derivative N‐hydroxy‐pipecolic acid (Chen et al., 2018; Hartmann et al., 2018). Initial research suggested that salicylic acid (SA) is a mobile signal that induces SAR (Malamy, Carr, Klessig, & Raskin, 1990), but later studies indicate that rather than being transported from infected areas, SA biosynthesis increases in distal tissues (Molders, Buchala, & Metraux, 1996; Vernooij et al., 1994). Currently, AzA is widely accepted as a mobile systemic defence signal, and SA and pipecolic acid are regarded as systemic amplifiers of SAR signals (Bernsdorff et al., 2016). In addition, lipid transfer proteins (LTPs), azaleic acid induced 1 (AZI1), and defective in induced resistance (DIR1) are required for SAR responses to bacterial infection (Jung et al., 2009; Maldonado, Doerner, Dixon, Lamb, & Cameron, 2002).

In this study, we investigated the effect of bacterial infection on stomatal development, using the stomata‐entering pathogen *Pst*DC3000 in the model species Arabidopsis thaliana. We report that SD, but not SI, is reduced in new leaves due to increased epidermal cell size and that the molecular mechanism responsible for this SD response requires perception by the bacterial flagellin receptor and endogenous SA production. We investigated whether a reduction in the density of stomata could affect pathogenesis. To do this, we infected Arabidopsis stomatal development mutants with *Pst*DC3000 and demonstrated that leaf SD influences pathogen colonization. We therefore propose that a reduction in stomatal entry points following pathogen infection could aid in protecting plants against diseases caused by foliar pathogens.

## 2 MATERIALS AND METHODS

### 2.1 Plant growth

A. thaliana plants (Col‐0 and mutant genotypes derived from this accession) were grown under short‐day conditions in a controlled environment chamber (9 hr day^−1^ in length, 200 μmol·m^−2^·s^−1^ of light, at 22°C day^−1^, 18°C night^−1^). Seed were vernalized for 2 days at 4°C and plants grown on 44 mm of Jiffy‐7 peat pellets (Jiffy Products International AS, Norway). Isolation and characterization of *ald1‐1* (SALK_007673), *azi1‐1* (Jung et al., 2009), NahG (van Wees & Glazebrook, 2003), *nced3 nced5* (Frey et al., 2012), *fls2C* (SAIL_450_D03; Gómez‐Gómez & Boller, 2000), *sid2‐1* (Nawrath & Métraux, 1999), *npr1‐1* (Cao, Bowling, Gordon, & Dong, 1994), and *dir1‐1* (Maldonado et al., 2002) have been previously described. SD mutants *EPF2*OE, *EPFL9*OE, and *basltmm* have been described previously (Hunt, Bailey, & Gray, 2010; Hunt & Gray, 2009; Hunt & Gray, 2010). *EPFL7* overexpressing plants were created by amplifying the *EPFL7* gene from Arabidopsis genomic DNA with primers cacctttcttttgttcaaaaccctttc and cacgtgagatgaggaaggatt, combining into Pentr/d/TOPO and recombining into pCTAPi (Rohila, Chen, Cerny, & Fromm, 2004) with LR Clonase II. *EPFL7‐CTAPi* was then transformed into Agrobacterium strain C58 by electroporation, transformed into A. thaliana via floral dipping (Clough & Bent, 1998) and selected by spraying with Basta (Liberty Agrevo). Antibiotics kanamycin and rifampicin were used at 50 μg ml^−1^ where required.

### 2.2 Bacterial challenge and leaf extraction

*P. syringae* pv. *tomato* DC3000 and green fluorescent protein (GFP)‐expressing derivative (Yu et al., 2013) were grown overnight at 28°C in 250 ml of Kings B medium supplemented with rifampicin. Cultures were centrifuged and pellets resuspended in 10 mM of MgSO_4_.

Three leaves of 5‐week‐old plants were syringe infiltrated with 0.2 ml of bacteria at an OD_600_ of 0.01 suspended in 10 mM of MgSO_4_. At the time of inoculation, the youngest visible leaves at the centre of the rosette were marked with small dots of acrylic paint. Control plants were mock syringe inoculated with 10 mM of MgSO_4_. Leaf epidermal cell counting was carried out on leaves that developed after the marked leaves when they had fully expanded. ImpressPLUS dental resin (Perfection Plus Ltd, Hants) was applied to the abaxial surface of fully developed leaves and nail varnish peels were taken from the set resin after removing the leaf. SD counts were taken from three 0.25 mm^2^ of areas of leaves on eight separate plants of each genotype. Epidermal cell size measurements were taken from three areas of leaves on five separate plants that had undergone each treatment. Images of resin peels (described above) were taken under bright field microscopy under ×20 magnification and were analysed using ImageJ software. Each epidermal pavement cell shape was traced before measurements were taken. Guard cell length and width were measured and analysed under ×40 magnification using ImageJ software from the resin peels of five plants with a minimum of 50 individual guard cells being measured per treatment. Stomatal complex area was calculated using the equation π (length/2) * (width/2) assuming the area is elliptical.

For dip inoculation, the whole leaf rosette of each plant was submerged and gently swirled for 5 s in a bacterial suspension in 10 mM of MgSO4 adjusted to OD_600_ 0.2 and supplemented with 0.002% Silwet L‐77. Plants were immediately placed in an incubator to maintain high humidity. Using a leaf borer, 28.33 mm^2^ of leaf disks were taken, washed gently for 10 s in 70% ethanol, and further rinsed in sterile 10 mM of MgSO_4_ for 30 s. Leaf disks were macerated in Eppendorf tubes containing 500 μl of sterile MgSO_4_ with a micropestle, vortexed, and serially diluted before plating onto Kings B agar medium supplemented with antibiotics.

GFP‐tagged bacteria were imaged using a Leica DXM‐6 light florescence microscope. Leaves of SD mutants were incubated for 12 hr in Petri dishes with OD_600_ 0.2 bacterial cultures at 22°C in the light prior to imaging.

### 2.3 Chlorophyll fluorescence analysis

Six‐week‐old plants were dip‐inoculated with *P. syringae pv*. DC3000 in 10 mM of MgSO_4_ with 0.02% Silwet L‐77 adjusted to OD_600_ 0.2 or mock‐inoculated (10 mM of MgSO_4_, 0.02% Silwet L‐77). Dark‐adapted *F*_v_/*F*_m_ was measured by imaging whole rosettes before inoculation and at 24, 30, 36, and 48 hr post inoculation with the Closed FluorCam FC 800‐C (Photon Systems Instruments) using the manufacturer's protocol with the *f* settings: excitation light Act1 and Super at 70% of the respective maximal values, 34% sensitivity and 20 μs of shutter speed. Image analysis was carried out with the FluorCam7 software (Photon Systems Instruments). *F*_v_/*F*_m_ values over the whole rosettes were extracted and percentage leaf area with *F*_v_/*F*_m_ values below 0.7 was calculated.

### 2.4 Elicitor treatment

Flg22 peptide and lipopolysaccharide (LPS) purified from Pseudomonas aeruginosa serotype 10 were diluted in 10 mM of MgSO_4_ to final concentrations of 200 nM of flg22 (synthesized by Source Bioscience), and 100 μg ml^−1^ LPS and L‐pipecolic and AzA (Sigma‐Aldrich) were dissolved in 5 mM of MES before syringe infiltration as above. Control “mock‐treated” plants were infiltrated with 10 mM of MgSO_4_ or 5 mM of 2‐(N‐morpholino)ethanesulfonic acid (MES) buffer as appropriate.

### 2.5 Estimation of transcript levels

RNA was extracted using a Spectrum™ Plant Total RNA Kit (Sigma‐Aldrich), and cDNA was synthesized with Maxima H Minus Reverse Transcriptase (Thermo Scientific) following the manufacturer's guidelines. Developing leaves from four individual plants were pooled together to generate each sample with three samples being used per treatment group. Ten microlitre volume quantitative PCR reactions were carried out using a Corbett Rotor Gene 6000. Each reaction contained 5 μl of 2× Rotor‐Gene® SYBR® Green PCR master‐mix, 0.25 μl of forward and reverse primers, and 8 ng of cDNA. Relative transcript quantities were calculated according to (1 + E)^ΔCt^, where ΔCt = Ct (sample) − Ct (calibrator sample), and normalized to (1 + E)^ΔCt^ values of two reference genes *At2g28390* and *At5g25760*. Primer sequences designed to amplify 3′ end of each transcript were as published (Casson & Hetherington, 2014; Czechowski, Stitt, Altmann, Udvardi, & Scheible, 2005).

### 2.6 Infection with Hyaloperonospora arabidopsidis

Leaves were infected by spraying condiospores of *H. arabidopsidis* (*Hpa*) WAC09 onto 10‐day‐old plants at a density of 10^6^ spores per millilitres. The colonization of this oomycete infection was analysed in whole leaves stained with lactophenol‐trypan blue (200 mg L of 1Trypan blue, 100 mg L of 1 phenol, 10% glycerol, 10% *v*/v lactic acid, and 60% ethanol), boiled for 1 min in trypan blue solution, and placed in chloral hydrate to decolourise, as described by Koch & Slusarenko, 1990. Fifty leaves of each genotype were analysed under a compound light microscope, and the level of colonization by the pathogen was assigned to one of four classes as defined by Luna, Bruce, Roberts, Flors, and Ton (2012): Class I, no pathogen growth; Class II, hyphal colonization without conidiospores; Class III, hyphal colonization with conidiospores and sporadic oospores; Class IV, extensive colonization, conidiospores, and frequent oospores.

## 3 RESULTS

### 3.1 Pathogen infection systemically reduces SD in new leaves

**Figure 1:**
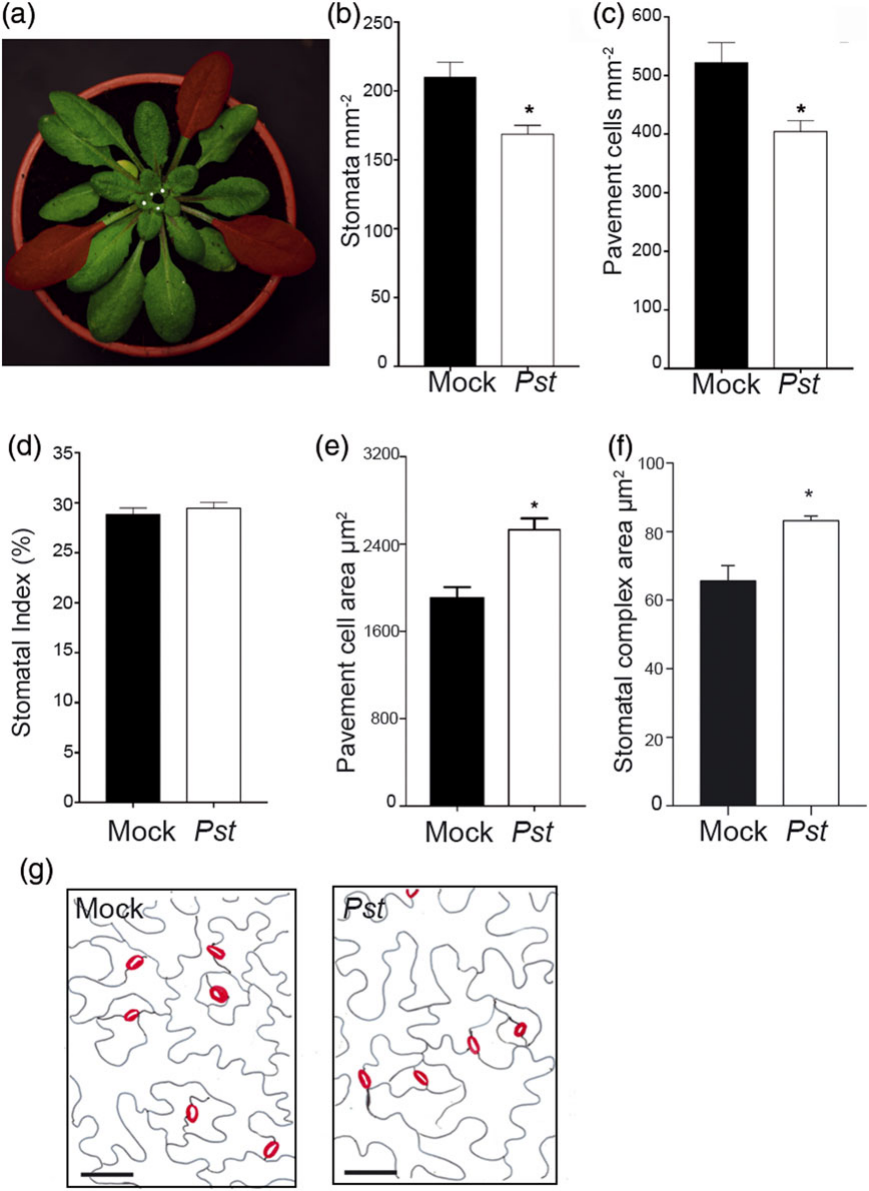
Stomatal density is reduced following bacterial infection. (a) Experimental set‐up: Three mature Arabidopsis leaves (in red) were infiltrated with PstDC3000 or mock treated. The youngest leaves present at the time of infection were marked with white dots. When the next leaves to emerge at the centre of the rosette (area marked in black) had fully expanded and were mature, their epidermal cell densities and sizes were analysed. (b) Mean stomatal density, (c) epidermal pavement cell density, (d) stomatal index, and (e) epidermal pavement cell and (f) stomatal complex size of new leaves developed after mock treatment or infection with PstDC3000. *Significant difference in comparison with mock treatment (Student's t test, p < .05, n = 8 plants, except for panels e and f, n = 5). Error bars = SE. Tracings of the epidermis of representative leaves from this experiment are shown in (g)

To understand the effect of bacterial infection on stomatal development, three mature leaves of 5‐week‐old Arabidopsis plants were infiltrated with *Pst*DC3000. At the time of infiltration, the youngest emerging leaves were marked (Figure 1a). Plants were grown for approximately 3 weeks following inoculation until the next leaves emerged (after the marked leaves) had fully expanded, and then SD and SI were determined from these newly developed leaves. Plants inoculated with *Pst*DC3000 showed a significantly reduced SD on their new leaves (169 mm^−2^) in comparison with mock‐inoculated plants (210 mm^−2^; Figure 1b). Hence, in this experiment, localized *Pst*DC3000 infection on mature leaves reduced SD by 20% in subsequently developing leaves. This experiment was repeated on seven occasions with similar results (Table S1). Reductions in SD ranged between 5.7% and 20% and were significant in the majority of experiments. Epidermal pavement cell density was also reduced by 22% (from 522 to 405 mm^−2^), in new leaves following infection (Figure 1c). Mean SI values did not differ between mock‐ and *Pst*DC3000‐inoculated plants (Figure 1d). The size of both epidermal pavement cells and stomata increased following infection (by 32% and 27%, respectively; Figure 1e,f). We did not observe additional arrested stomatal precursor cells in leaves following infection (Figure 1g).

The leaves directly infiltrated with *Pst*DC3000 wilted and died over a period of several days, whereas mock‐inoculated leaves remained unaffected. To confirm that the reduced SD response in new leaves was not due to reduced plant photosynthetic capacity, three similarly sized mature leaves were excised from uninfected plants, and the next emerging leaves of these plants were examined 3 weeks later. Leaf removal had no effect on SD of newly developed leaves (Figure S1A). Furthermore, following infection of mature leaves, we failed to extract *Pst*DC3000 bacteria from newly developing leaves (Figure S1B). Together, these results indicate that mature *Arabidopsis* leaves infected by *Pst*DC3000 produce a systemic signal that adjusts epidermal development in newly emerging leaves, resulting in reduced density of stomatal pores through production of fewer larger epidermal cells.

### 3.2 PstDC3000‐induced reduction in SD requires bacterial PAMP perception and SA biosynthesis

**Figure 2:**
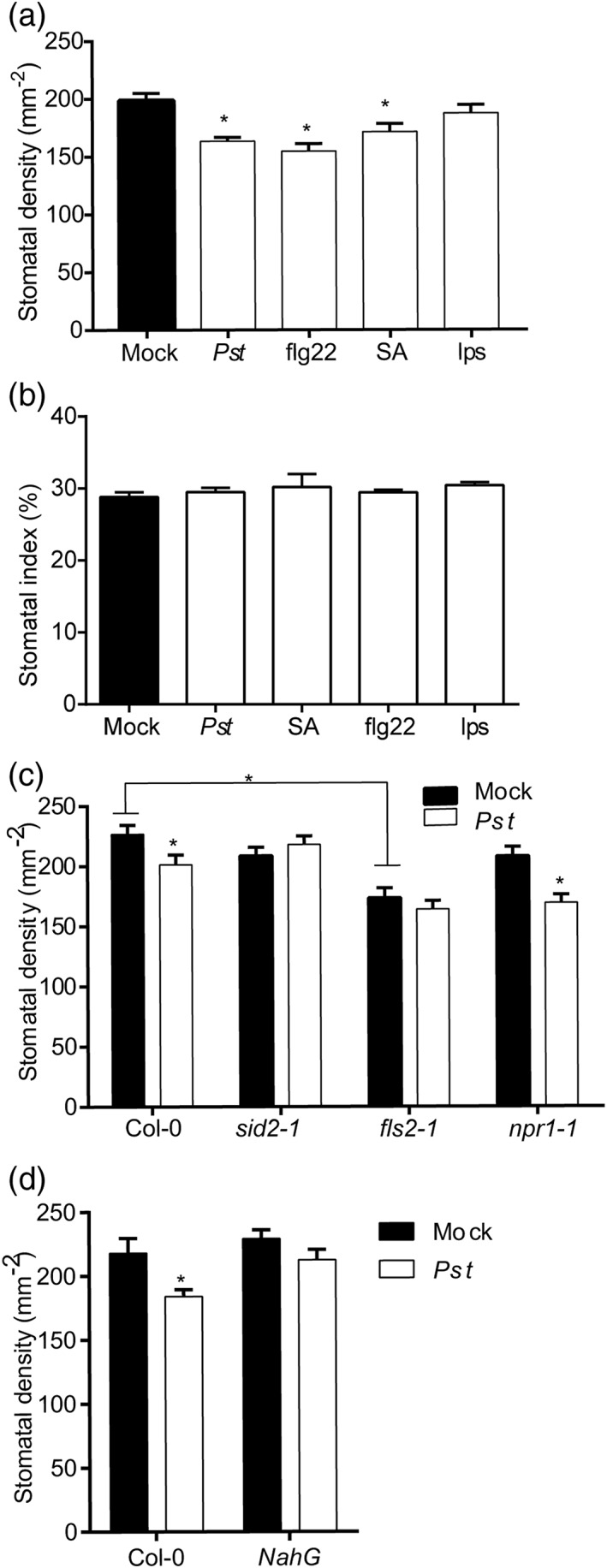
Bacterial‐induced reductions in stomatal density require flagellin perception and salicylic acid biosynthesis and metabolism. Mean (a) stomatal densities and (b) index of leaves developed after infiltration with PstDC3000 or elicitors flg22, SA, and LPS. Mean stomatal densities of defence signalling mutants (c) sid2‐1, fls2C, npr1‐1, and (d) NahG and Col‐0 control plants after infiltration with PstDC3000. *Represents a significant difference in comparison with mock treatment (Student's t test, p < .05, n = 8 plants) or a significant difference between stomatal genotypes with two‐way analysis of variance and Tukey's post hoc test (p < .05, n = 8 plants). Error bars = SE

To examine the molecular nature of the signals involved in the reduced SD response, we infiltrated mature Arabidopsis leaves with well‐characterized chemical elicitors of SAR and quantified SD in next emerging leaves. Infiltration with either 200 nM of the pathogen‐associated molecular pattern (PAMP) flg22 or 1 mM of SA induced a systemic reduction in SD that was comparable with that of *Pst*DC3000‐inoculated plants. In the experiment presented in Figure 2a,b, *Pst*DC3000 infection reduced SD by 18%, flg22 by 22%, and SA by 13%, but SI was not affected. However, infiltration of mature leaves with LPS PAMP (Zeidler et al., 2004) failed to induce a statistically significant SD response. Repeating these experiments gave similar results (Figure S2).

To further test the role of flg22 in the systemic SD response, we carried out experiments with Arabidopsis mutant plants that are affected in the FLS2 flagellin receptor (Zipfel et al., 2004). Bacterial infiltration of *fls2C* plants did not result in a statistically significant reduction in SD, indicating that both flagellin and its receptor are involved in the SD response to *Pst*DC3000. Notably, *fls2C* had significantly lower SD than Col‐0 with or without infection (Figure 2c), which has not been previously reported and was not further studied here. To determine the role of SA production, we challenged *sid2‐1* plants, which are impaired in their ability to produce SA upon pathogen infection (Wildermuth, Dewdney, Wu, & Ausubel, 2001), *npr1‐1* plants, defective in the NPR1/NIM1 protein function that regulates SA‐dependent defence gene induction (Cao et al., 1994), and *NahG* plants, which convert SA into catechol (Lawton et al., 1995). Neither *sid2‐1* nor *NahG* was able to reduce SD following infection (Figure 2c–d), supporting a role for SA in the systemic SD response to *Pst*DC3000. However, *npr1‐1* plants retained the ability to reduce SD following infection, suggesting that the systemic SD response is mediated by NPR1‐independent SA signalling.

### 3.3 Systemic reductions in SD require AZI1 but not L‐pip, DIR1, and ABA

**Figure 3:**
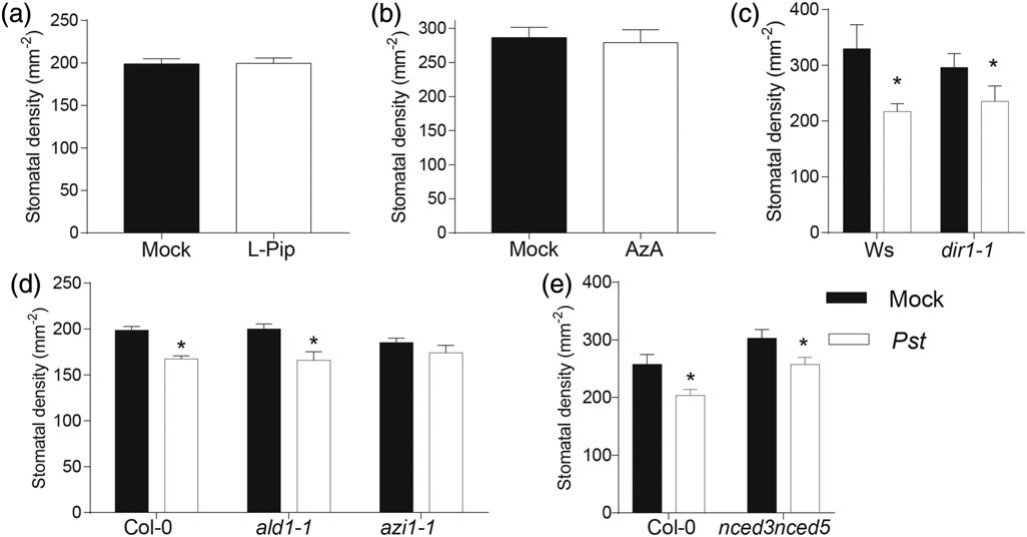
Systemic defence component AZI1 is involved in systemically reducing stomatal density. (a) Mean stomatal densities of leaves developed after 100 μg ml^−1^ of pipecolic acid (L‐pip) infiltration. (b) Mean stomatal densities of leaves developed after 1 mM of azelaic acid (AzA) infiltration. Mean stomatal densities of (c) dir1‐1, (d) ald1‐1, and azi1‐1, and (e) nced3 nced5 systemic signalling mutants leaves developed after syringe infiltration with PstDC3000 in comparison with Col‐0 or Ws as controls. *Significant difference in comparison with mock treatment (Student's t test, p < .05, n = 8 plants). Error bars = SE

To investigate potential systemic signals involved in the pathogen‐induced SD response*,* we infiltrated Arabidopsis with chemicals involved in transmitting other long distance signals from infected leaves to uninfected areas of the plant and studied the systemic SD response in respective mutants. The pipecolic acid (L‐pip) deficient *agd2‐like defence response protein1* mutant (*ald1‐1*) has compromised SAR (Song, Lu, McDowell, & Greenberg, 2004), but infiltration of Col‐0 with L‐pip did not alter SD in newly developed leaves after *Pst*DC3000 inoculation (Figure 3a). The systemic SD response was intact in the *ald1‐1* mutant (Figure 3d), suggesting that pipecolic acid is not involved in this pathway. We infiltrated Col‐0 leaves with 1 mM of AzA, which is sufficient to elicit SAR (Jung et al., 2009), and assayed newly developed leaves for systemic SD reduction. Localized infiltration with AzA failed to reduce SD levels in new leaves (Figure 3b). In addition, *dir1‐1* plants, which lack an LTP involved in long‐distance regulation of SAR (Maldonado et al., 2002), were still able to reduce SD (Figure 3c). However, AzA‐insensitive *azi1‐1* plants, which are impaired in a different LTP (Jung et al., 2009), failed to show a significant SD response to localized *Pst*DC3000 inoculation (Figure 3d). This suggests that AZI may be involved in transmission of the systemic SD response but that its role is not directly related to the presence of AzA. However, our experiments used a relatively high concentration of AzA, and it remains possible that a different concentration of AzA may be effective. Abscisic acid (ABA) has also been implicated in long distance signalling in plants (Suzuki et al., 2013), but *nced3 nced5* plants, which produce much reduced levels of ABA (Frey et al., 2012), were unaffected in the systemic SD response to *Pst*DC3000 (Figure 3e). Thus, L‐pip, AzA, DIR1, and ABA do not appear to act as critical long‐distance signals in in the systemic SD response.

### 3.4 Pathogen‐induced reductions in SD are not associated with altered expression of genes known to control stomatal development

To determine a possible role for known regulatory components in stomatal development, we profiled systemic transcription of genes encoding epidermal patterning factor (EPF) peptides and the bHLH transcription factors SPEECHLESS and MUTE at 24 and 48 hr after localized *Pst*DC3000 infection. No statistically significant differences in transcript levels of these genes could be detected in developing tissues at the centre of the Arabidopsis rosette (Figure S3). This result is in line with our finding that *Pst*DC3000 affects SD but not SI (Figure 1b,d), suggesting that the observed decrease in pavement cell density may be regulated by a different developmental pathway that influences epidermal cell size. However, the extremely restricted temporal and spatial expression patterns of the genes tested make it difficult to exclude them from regulating pathogen‐induced alterations in SD.

### 3.5 SD affects leaf colonization by a bacterial pathogen

**Figure 4:**
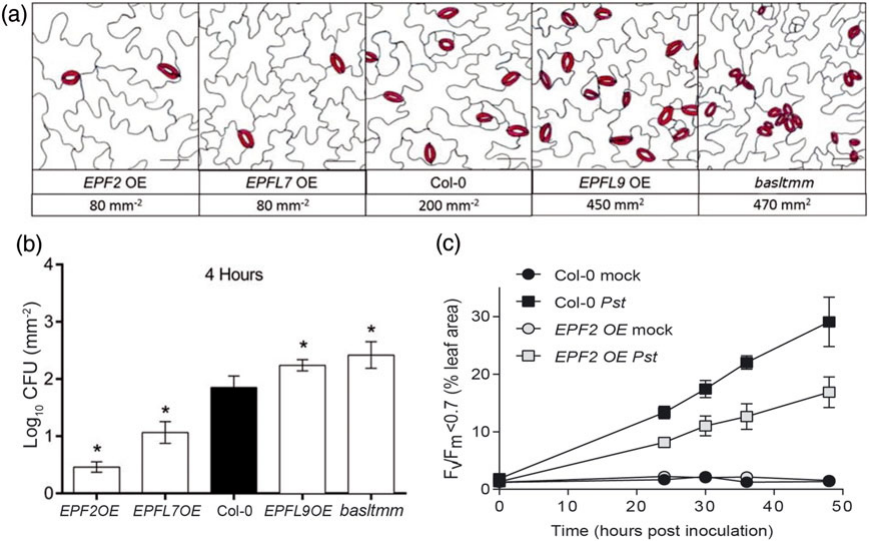
Effect of stomatal density on bacterial colonization. (a) Tracings of epidermis of Arabidopsis stomatal density mutants (stomata indicated in red). Genotypes and mean stomatal densities of fully expanded leaves are indicated below. (b) Colony‐forming unit (CFU) titres of leaf extracts 4 hr after dip inoculation with PstDC3000. *Significant differences from Col‐0 (Student's t test, p < .05, n = 8 plants). (c) Leaf rosette area with dark adapted F_v_/F_m_ chlorophyll fluorescence measurement of less than 0.7 (a level of photosynthetic efficiency indicative of severe stress) taken from 0 to 48 hr of postinoculation. Bars are SE. *Significant differences between PstDC3000‐treated Col‐0 and EPF2 OE plants (two‐way analysis of variance with Tukey's post hoc test, p < .05, n = 5 plants) [Colour figure can be viewed at wileyonlinelibrary.com]

The results described above suggest that plants might reduce the density of stomata that develop following infection, so that their new leaves would be more resistant to a subsequent bacterial attack. However, it would be difficult to test whether these new leaves have enhanced resistance as other systemic defence priming responses could also have been initiated. These would complicate any experiments and make it impossible to distinguish whether any observed decrease in bacterial colonization following a secondary infection was due to defence priming or the reduction in SD. Therefore, to explore why plants might develop fewer stomata following bacterial infection, we took a different approach and investigated whether artificially altering SD could affect bacterial infection. To do this, we quantified the level of infection in Arabidopsis mutant genotypes with altered levels of EPF peptides and associated signalling components that regulate stomatal development. It should be noted that these mutants display a greater range in SD than we observed in newly formed leaves of wild‐type plants following *Pst*DC3000 infection of mature leaves, with mutant SD ranging from 40% to 235% of Col‐0 (Figure 4a). Unlike the previous experiments, plants were dip‐inoculated with *Pst*DC3000 to allow colonization through the stomata. At 4 hr of postinoculation (hpi), the number of colony‐forming units showed a positive relationship with SD (Figure 4b). Mutants with decreased SD allowed significantly lower levels of *Pst*DC3000 colonization, whereas those with increased SD had significantly higher levels of colonization. For example, *EPF2*‐overexpressing plants, which showed a 60% reduction in SD, allowed a 77% lower colonization level than Col‐0 controls. Conversely, *basltmm* with a >100% increase in SD sustained 87% more bacteria in its leaves. At 24 hpi, these differences in colonization level persisted in three out of four of the stomatal mutants (Figure S4A). We also investigated GFP‐tagged *Pst*DC3000 patterns of early leaf colonization in the same plant lines and confirmed that bacteria localized predominantly around the stomata. There were therefore fewer bacteria attached to the surface of leaves with reduced SD, and these were mainly associated with the rare stomatal complexes (Figure S4B). Conversely, there were greater numbers of bacteria colonizing leaves with increased SD. These results indicate that the frequency of stomata affects the ability of the pathogen to attach and colonize the phyllosphere of *Arabidopsis*.

To determine whether SD affects whole‐plant disease symptom development, we employed chlorophyll fluorescence imaging to quantify *F*_v_/*F*_m_ values as an early marker for disease symptom development (Berger et al., 2007). From 24 to 48 hpi, the area of leaves exhibiting severe stress (with dark‐adapted *F*_v_/*F*_m_ values below 0.7) was significantly smaller in *EPF2*‐overexpressing plants with reduced levels of SD (Figure 4c). The reduced *F*_v_/*F*_m_ values at 24 and 48 hpi correlated with visibly less severe bacterial speck symptom development at 72 hpi (Figure S4C), indicating that a reduction in SD can reduce disease symptom development.

We excluded the possibility that the attenuated disease symptoms observed in SD mutants are due to an alteration in postinvasive resistance, rather than stomatal (preinvasive) resistance. To do this, wild‐type plants were challenged with the oomycete pathogen *Hpa* WACO9, which does not infect through the stomata and is resisted by SA‐dependent postinvasive defences (Coates & Beynon, 2010), or were syringe infiltrated with *Pst*DC3000 to bypass stomatal resistance. Neither *EPF2* nor *EPFL9* overexpressing plants were altered in their basal resistance against *Hpa* (Figure S4D). Furthermore, we could not detect differences in levels of *Pst*DC3000 colonization following syringe infiltration of the leaves (Figure S4E). Hence, the observed differences in *Pst*DC3000 colonization after dip‐inoculation (Figure 4) are most likely due to the altered number of stomatal entry points rather than other changes in postinvasive defence.

## 4 DISCUSSION

In this study, we establish that localized infection with the bacterial leaf pathogen *Pst*DC3000 systemically supresses SD in leaves that subsequently develop. However, the relatively small reduction in SD in leaves following infection observed here would not be expected to detrimentally affect either photosynthetic or reproductive capacity of the plant. Although very substantial reductions in SD will affect photosynthetic rates, plants are not normally limited by their CO_2_ uptake capacity. Previously, plants manipulated to have reductions in SD that are more severe than the 20% reductions caused by infection (Figure 1) have shown little or no suppression in photosynthetic capacity or seed yield. For example, Arabidopsis *EPF2*‐overexpressing plants that have an 80% reduction in SD showed a small reduction in net photosynthesis at high light, but *EPFL9*‐RNAi plants with a 68% reduction in SD showed no significant differences (Franks, Doheny‐Adams, Britton‐Harper, & Gray, 2015; Tanaka, Sugano, Shimada, & Hara‐Nishimura, 2013), and barley plants overexpressing HvEPF1 with up to 58% reductions in SD showed no reductions in seed yield (Hughes et al., 2017).

We propose that bacterial infection generates a systemic signal that is translocated from the mature infected leaves to the developing leaves in the apical meristem, where it reduces SD by increasing epidermal cell expansion in the newly developing leaves (Figure 1). The lack of arrested stomatal precursor cells in leaves formed after infection, together with the production of larger epidermal cells, suggests that, rather than stomatal precursor cells becoming arrested, fewer epidermal cells enter the stomatal lineage during the early stages of leaf development. It has been previously reported that plant species, or mutants, or environmental perturbations, which produce a low density of stomata, often also produce larger stomata (e.g., Doheny‐Adams, Hunt, Franks, Beerling, & Gray, 2012). This inverse correlation between SD and size was observed here following bacterial infection (Figure 1).

The flg22 bacterial peptide was also able to reduce stomatal development in our experiments. Recent evidence shows that flg22 is itself potentially mobile (Jelenska, Davern, Standaert, Mirzadeh, & Greenberg, 2017), but its effect in distal tissues remains unknown and was not explored here. Instead, by studying Arabidopsis genotypes with defects in defence responses and by applying chemical defence elicitors, we have shown that the FLS2 flagellin receptor, SA production, and the AZI1 protein are most probably required to perceive the bacterial stress signal and to generate and/or perceive a yet unknown systemic developmental signal (Figures 2 and 3).

Perhaps surprisingly, our results suggest that this SA‐mediated response is regulated via an NPR1‐independent signalling pathway (Figure 2c). However, although NPR1 is generally regarded as a key positive regulator of SA responses (Yan & Dong, 2014), several other SA triggered pathogen responses have been reported to be NPR1 independent (Blanco et al., 2005; Janda & Ruelland, 2015; Uquillas, Letelier, Blanco, Jordana, & Holuigue, 2004), or even antagonistic (Li, Bonaldi, Uribe, & Pruneda‐Paz, 2018).

We also demonstrate that several SAR regulatory components, including pipecolic acid, AzA, ABA, and the bacterial PAMP LPS do not appear to be required for pathogen‐induced reductions in stomatal development (Figures 2 and 3). AzA is believed to be required for the regulation of SAR through its assumed interaction with the LTP, AZI1 (Jung et al., 2009). Our finding that AZI1 is necessary for the *Pst*DC3000‐induced systemic SD response, whereas AzA and another LTP DIR1 are probably not (Figure 3), suggests that AZI1 might mediate the systemic SD response through perception or transport of a different signalling molecule(s). Because the AzA precursor 9‐oxo nonanic acid is also a potential systemic signal (Wittek et al., 2014), it is possible that AZI1 mediates the transport of nonanic acid or a similar systemic lipid signalling molecule. AZI has also been described as a hybrid proline rich protein (Pitzschke, Xue, Persak, Datta, & Seifert, 2016), but the functional significance of this is unknown.

Our data support the interaction of epidermal cell patterning and defence pathways, but the exact nature of this interaction and its potential role in systemic SD responses remain to be characterized. Several previous observations indicate a link between the response to pathogen infection and the frequency of stomatal development. Indeed, expression of bacterial effectors in planta causes increased stomatal development (Meng et al., 2015), and our study reports that plants lacking the flagellin receptor FLS2 have constitutively lower levels of stomatal development (Figure 2c). Compromised immunity in plants deficient in ERECTA, a receptor‐like kinase involved in the regulation of stomatal development (Godiard et al., 2003; Llorente, Alonso‐Blanco, Sánchez‐Rodriguez, Jorda, & Molina, 2005), provides further support, and it seems probable that pathogen defence responses and the stomatal development pathway might converge through activation of shared mitogen‐activated protein kinase (MAPK) cascades. Defence gene induction through FLS2 and control of stomatal development require similar MAPK and MAPK kinase (MKK) activities (Asai et al., 2002; Wang, Ngwenyama, Liu, Walker, & Zhang, 2007), the MKKK YODA regulating stomatal patterning also affects pathogen resistance (Sopeña‐Torres et al., 2018; Sun et al., 2018), and it appears that pathway‐specific MKKKs may compete for shared MKKs in the pathways controlling stomatal development and immunity (Sun et al., 2018). However, despite this known interaction, we could not find transcriptional evidence to support the involvement of EPF or bHLH stomatal development regulators that act upstream and downstream of this MAPK cascade in systemic SD responses, and the direct involvement of MAPK components remains to be investigated. SA and jasmonic acid frequently act antagonistically to each other in defence responses; however, the reduction in SD seen in Figure 2c has similarities to that seen in cotyledons when jasmonic acid is applied (Han et al., 2018) suggesting further differences between the pathways involved in pathogen responsive gene induction and pathogen induced SD change.

Plants with closed stomatal pores (e.g., at elevated CO_2_ levels) are known to have enhanced resistance to bacterial pathogens (Li et al., 2015), but the pathological significance of reduced SD has not been thoroughly studied previously. Our experiments with stomatal development mutants revealed that the number of leaf‐colonizing bacteria is influenced by the number of stomatal entry points for at least 24 hr after inoculation. Furthermore, disease‐related symptoms in plants with fewer stomata were reduced for several days after inoculation, suggesting that a decrease in pathogen entry points gives the plant additional time to mount an appropriate defence response (Figure 4). Our findings are supported by the recent demonstration that reducing SD on the leaves of cultivars of *Gentiana trifolia* slows the rate of *Septoria gentianae* fungal infection, with leaves with the highest SD showing the greatest incidence of infection (Tateda et al., 2018). Our results indicate that plants may actively reduce stomatal development in newly developing leaves as a means to acclimate to bacterial disease pressure. Hence, stomatal immunity not only contributes to disease resistance through stomatal closure in local tissues at relatively early time points after infection (Melotto et al., 2006), it also contributes to a longer term mechanism for disease resistance through reducing the development of stomatal entry points in newly emerging leaves.

We have found that SA, FLS2, and AZI1 are required for a systemic reduction in SD in response to bacterial infection. The remaining molecular components of this pathway, and whether the systemic reduction in SD and/or SI found in response to virus infection (Murray et al., 2016) or abiotic triggers such as light, air humidity, and CO_2_ concentration (Lake et al., 2001; Schoch et al., 1980; Thomas et al., 2004; Woodward, 1987) employ similar mechanisms, remain to be characterized. Because many foliar pathogens, including several that cause substantial losses in cereal crops, gain access to their host through stomatal pores, our results raise the possibility of enhancing preinvasive resistance through creating crop cultivars with reduced numbers of stomata.

## FUNDING INFORMATION

J.E.G., J.T. and H.H. thank the Leverhulme Trust (RPG‐2016‐274), J.E.G. and L.H. thank the BBSRC (BB/N004167) for project grant funding, and C.D. thanks the Grantham Centre for Sustainable Futures for funding his PhD studentship.

## AUTHOR CONTRIBUTIONS

J.E.G., J.T., L.H., H.H. and C.D. designed research; L.H., H.H., C.H., A.M. and C.D. performed research; J.E.G., J.T., L.H., H.H. and C.D. analysed data; and J.E.G., J.T., H.H., L.H. and C.D. wrote the paper.

## Supporting information


**Table S1.** Related to Figure 1. Reductions in stomatal density were seen following infection by Pseudomonas syringae DC3000 across seven independent experiments.
**Figure S1.** Related to Figure 1. Bacterial‐induced systemic reduction in SD is not caused by loss of photosynthetic capacity, or movement of bacteria to developing leaves.
**Figure S2.** Related to Figure 2. Bacterial‐induced reductions in stomatal density require flagellin perception and salicylic acid accumulation.
**Figure S3.** Expression levels of stomatal development genes are not altered following bacterial infection.
**Figure S4.** Related to Figure 4. Stomatal density mutants have altered susceptibility to bacterial infection through the stomata but are not affected in basal resistance.
