# Supplementary material for: Bacterial infection systemically suppresses stomatal density
Source: Plant Cell Environ. 2019 Jun 10;42(8):2411–21. doi: 10.1111/pce.13570 (PMC6771828; doi:10.1111/pce.13570)
Supplement: Supplementary file 1 — Table S1. Related to Figure 1. Reductions in stomatal density were seen following infection by Pseudomonas syringae DC3000 across seven independent experiments. Figure S1. Related to Figure 1. Bacterial‐induced systemic reduction in SD is not caused by loss of photosynthetic capacity, or movement of bacteria to developing leaves. Figure S2. Related to Figure 2. Bacterial‐induced reductions in stomatal density require flagellin perception and salicylic acid accumulation. Figure S3. Expression levels of stomatal development genes are not altered following bacterial infection. Figure S4. Related to Figure 4. Stomatal density mutants have altered susceptibility to bacterial infection through the stomata but are not affected in basal resistance. [file PCE-42-2411-s001.docx]

**Supplemental Information**

**Bacterial infection systemically suppresses stomatal density**

**Christian Dutton, Hanna Hõrak, Christopher Hepworth, Alice Mitchell, Jurriaan Ton, Lee Hunt, and Julie E. Gray**

| Mock Infected (Stomata mm^-2^) | *Pst*DC3000  (Stomata mm^-2^) | % reduction  in density | P-value |
| --- | --- | --- | --- |
| 215.6 | 184.0 | 14.7 | 0.0356 |
| 198.7 | 163.3 | 17.8 | <0.0001 |
| 284.0 | 268.0 | 5.7 | 0.534 |
| 253.0 | 210.0 | 20.0 | <0.0001 |
| 226.7 | 201.3 | 11.1 | 0.0396 |
| 198.7 | 167.3 | 15.6 | <0.0001 |
| 258.7 | 234.0 | 9.5 | 0.0023 |

**Table S1.** **Related to Figure 1. Reductions in stomatal density were seen following infection by *Pseudomonas syringae* DC3000 across seven independent experiments.**

Comparison of stomatal densities of subsequently developing leaves from *Pst*DC3000 infected or mock inoculated Col-0 Arabidopsis plants (Student’s t-test for statistical analysis).

**B**


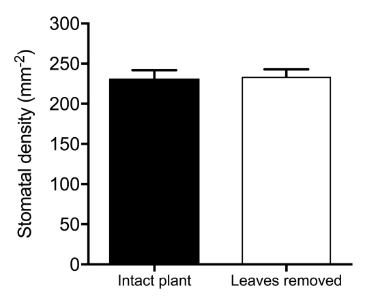

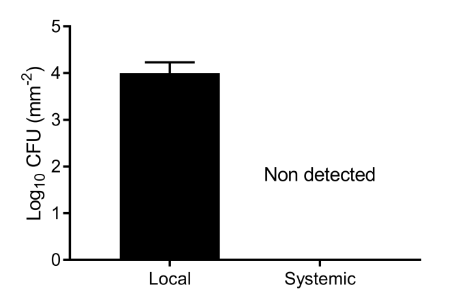


**A**

**Figure S1. Related to Figure 1. Bacterial-induced systemic reduction in SD is not caused by loss of photosynthetic capacity, or movement of bacteria to developing leaves.**

(A) Mean stomatal density of subsequently developing leaves on plants that had 3 leaves removed, compared with untreated plants. (B) Colony forming units of *Pst*DC3000 detected form the developing rosette and from the infected leaves following syringe infiltration. No statistically significant differences between treatment and control tissues in (A) were detected by Student’s t-test, α=0.05. n=8. Error bars = SE.


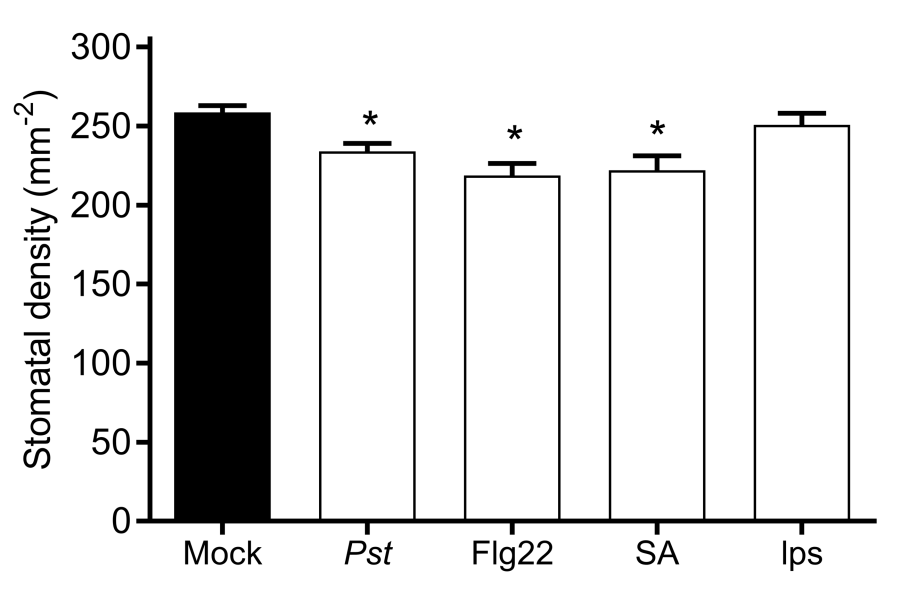


**Figure S2. Related to Figure 2. Bacterial-induced reductions in stomatal density require flagellin perception and salicylic acid accumulation.**

Mean stomatal densities of leaves developed after infiltration with *Pst*DC3000 or elicitors flg22, SA or LPS. *Significant difference in comparison to mock treatment (Student’s t-test, p<0.05, n=8 plants). Replicate experiment using independently grown plants.


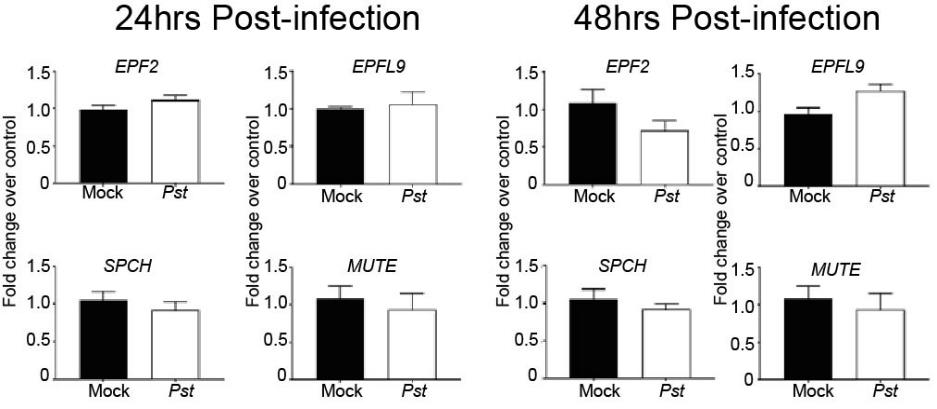


**Figure S3.** **Expression levels of stomatal development genes are not altered following bacterial infection.**

Rt-qPCR of RNA extracted from developing leaves in the rosette centre following infection of mature leaves with *Pst*DC3000. Expression levels of the Arabidopsis stomatal development genes *EPF2*, *EPFL9*, *SPEECHLESS* and *MUTE* at 24 hours and 48 hours post inoculation. Each point represents tissue pooled from 4 plants, repeated 3 times and was compared to control with unpaired t-test. Bars are SE


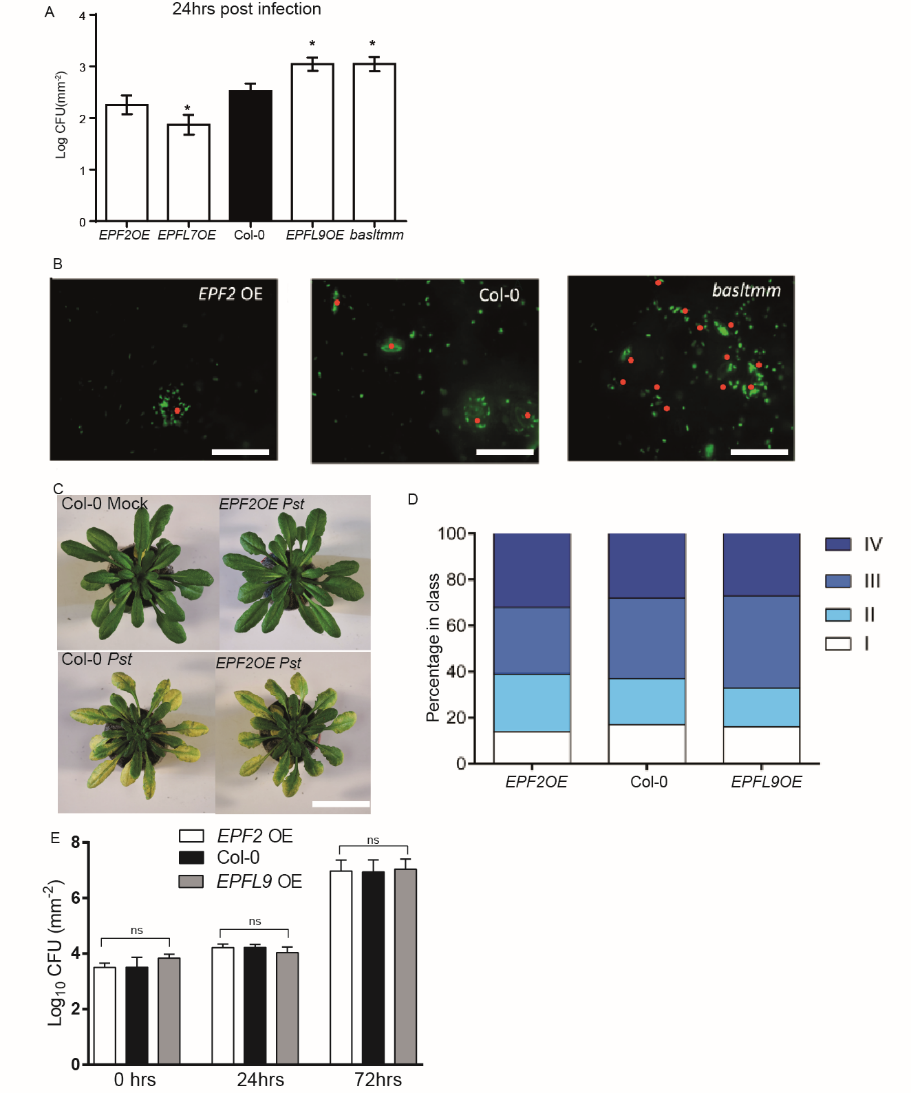


**Figure S4.** **Related to Figure 4.** **Stomatal density mutants have altered susceptibility to bacterial infection through the stomata but are not affected in basal resistance.**

(A) Colony forming unit (CFU) titres of leaf extracts 24 hours after being dip inoculated with *Pst*DC3000. Bars are SE. * denotes significant differences from wild-type plants (Student’s t-test, p<0.05, n=8 plants). (B) Fluorescent images of GFP-tagged PstDC3000 12 hrs after inoculation of mutant genotypes as indicated, red dots represent the position of stomatal pores. Scale bars represent 20 μm. (C) Images of representative plants from Figure 4C taken at 72 hours post inoculation with *Pst*DC3000. Scale bar = 5cm. (D & E) Basal levels of infection in stomatal density mutants EPF2OE and EPFL9OE were compared to Col-0 background plants. (D) Infection levels of *H.arabidopsidis* 6 days after conidiospore inoculation. Stained leaves were assigned to four infection classes based on Class I representing no visible infection and Class IV representing extensive levels of infection. Chi squared test revealed no significant distribution of classes between plant genotypes. (χ^2^ test; α= 0.05) comparing class distribution of mutants with Col-0 plants. (E) Bacteria were extracted from leaf discs 0 hrs, 24 hrs and 72 hrs after syringe infiltration of *Pst*DC3000. Bars are SE. No significant differences in extractable colony forming units (CFU) were observed between plant genotypes (Student’s t-test, α=0.05).
